# Swim-Rep fusion net: A new backbone with Faster Recurrent Criss Cross Polarized Attention

**DOI:** 10.1371/journal.pone.0321270

**Published:** 2025-05-27

**Authors:** Zhe Li

**Affiliations:** Wuhan University of Engineering Science, Wuhan City, Hubei Province, China; Universiti Tunku Abdul Rahman, MALAYSIA

## Abstract

Deep learning techniques are widely used in the field of medicine and image classification. In past studies, SwimTransformer and RepVGG are very efficient and classical deep learning models. Multi-scale feature fusion and attention mechanisms are effective means to enhance the performance of deep learning models. In this paper, we introduce a novel Swim-Rep fusion network, along with a new multi-scale feature fusion module called multi-scale strip pooling fusion module(MPF) and a new attention module called Faster Recurrent Criss Cross Polarized Attention (FRCPA), both of which excel at extracting multi-dimensional cross-attention and fine-grained features. Our fully supervised model achieved an impressive accuracy of 99.82% on the MIT-BIH database, outperforming the ViT model classifier by 0.12%. Additionally, our semi-supervised model demonstrated strong performance, achieving 98.4% accuracy on the validation set. Experimental results on the remote sensing image classification dataset RSSCN7 demonstrate that our new base model achieves a classification accuracy of 92.5%, which is 8.57% better than the classification performance of swim-transformer-base and 12.9% better than that of RepVGG-base, and increasing the depth of the module yields superior performance.

## Introduction

Swin Transformer is a hierarchical vision transformer that leverages shifted windows to efficiently process visual data. This architecture addresses key challenges in CV, such as high-resolution images and varying scales of visual entities. By computing self-attention within local windows and enabling cross-window connections through shifting, the Swin Transformer achieves linear computational complexity relative to image size. RepVGG is a variant of the VGG architecture that focuses on improving the efficiency and performance of convolutional neural networks (CNNs) for CV tasks. It combines the simplicity of VGG with the efficiency of modern CNNs, making it suitable for real-time applications.

In recent years, the development of multi-scale feature fusion modules has significantly advanced the field of computer vision, particularly in tasks such as image segmentation, object detection, and semantic understanding. These modules aim to effectively integrate features from different scales to capture both detailed and contextual information, thereby enhancing model performance. The TIF module was introduced in the DS-TransUNet architecture, designed for medical image segmentation [1]. This module leverages the Transformer’s self-attention mechanism to fuse features from dual-scale encoders, which extract both coarse and fine-grained features. By establishing global dependencies between multi-scale features, TIF ensures semantic consistency and has demonstrated superior performance in various segmentation tasks, especially in polyp segmentation. The MSFFM was proposed to address the challenge of semantic gaps in multi-scale feature fusion [2]. This module calculates pixel-wise correlations between different feature maps through matrix multiplication and uses these correlations as weight vectors for higher-level feature maps. MSFFM has been particularly effective in improving segmentation performance near object boundaries, such as in road pothole detection. The MSAA module was introduced as part of the CM-UNet architecture for remote sensing image segmentation [3]. It integrates spatial and channel attention mechanisms to enhance the representation of multi-scale features. MSAA replaces traditional skip connections in U-Net architectures, providing a more robust feature fusion mechanism. This module has shown significant improvements in capturing long-range dependencies and enhancing segmentation accuracy. The CEDNet is designed for dense prediction tasks [4]. This network structure focuses on better integrating multi-scale features by cascading encoder-decoder modules. It ensures that high-level features from early stages guide the learning of lower-level features, thereby enhancing the overall performance in tasks such as image segmentation.

In the past five years, attention mechanisms have continued to evolve and play a crucial role in advancing the field of computer vision (CV). These mechanisms help models focus on the most relevant parts of an image, thereby improving performance and efficiency. EANet was proposed, offering a novel approach to attention by using two linear layers to generate attention maps [5]. Unlike traditional self-attention mechanisms, EANet focuses on external interactions between features, which can be more efficient and effective in certain scenarios. GCT was introduced as a method to transform channels using a gated mechanism [6]. It normalizes the input features and applies a channel-wise normalization to generate attention weights, which are then used to modulate the input features. ECANet was proposed as an efficient channel attention mechanism [7]. It replaces the fully connected layers in SENet with a 1D convolution to reduce computational cost while maintaining performance. FcaNet was introduced, focusing on capturing multi-spectral channel attention [8]. It uses Discrete Cosine Transform (DCT) to analyze the frequency components of the feature maps, providing a more powerful representation than traditional methods. GLTR was proposed for video person re-identification [9]. It combines global and local temporal information using dilated 1D convolutions and self-attention mechanisms to capture both short-term and long-term dependencies.

Yan Gao’s research developed a Swin Transformer-based network for medical image segmentation that incorporates an inductive bias capability to improve accuracy. On the Synapse dataset, the DSC of Swin-IBNet surpasses that of Swin-Unet by 3.45% [10]. Huanshuo Zhang’s study explores the use of Swin Transformer and federated learning for plant leaf disease diagnosis, demonstrating its potential for application in agriculture, with an accuracy achieves 97.2% [11]. Ali Hatamizadeh’s research developed a Swin Transformer-based network (Swin UNETR) for semantic segmentation of brain tumours in MRI images, which combines the powerful feature extraction capability of Swin Transformer with the efficient segmentation capability of UNet [12].

The characteristics of these algorithms are often one-dimensional and fail to effectively integrate both global and local characteristics. In this study, our fusion model leverages the multi-scale characteristics of both the Swim Transformer and RepVGG architectures. Additionally, we integrate the Faster Recurrent Criss-Cross polarized Attention (FRCPA) module to filter and enhance these characteristics. The aim is to harness the strengths of both the Transformer and CNN architectures to yield more robust classification features.

The contributions of this paper are as follows:

1) We propose a novel multi-scale feature fusion module called MPF that uses a multi-branch strip-pooling structure to extract global features and fuse channel-attention local features to improve the feature representation of the model.
2) We introduce the FRCPA module, which utilizes the cross-crossing algorithm to extract attention from both horizontal and vertical perspectives. This, combined with the polarization attention algorithm, enables capturing fine-grained features.
3) Based on the structure of RepVGG and SwimTransformer, we propose a new backbone, Swim-Rep Net, which obtains the advantages of CNN and Transformer.
4) We conducted fully supervised modeling experiments on the MIT-BIH dataset and RSSCN7 dataset, as well as performance comparison experiments on the MPF module and FRCPA module. Finally, we tested the classification performance of the model in a semi-supervised state using pseudo-labeling semi-supervised technique on the MIT-BIH dataset.

## Related work

### Swim transformer and RepVGG

The swim-transformer method [13] represents a highly evolved variant of Vision Transformer that presents two notable advantages. Firstly, it incorporates gradually increasing downsampling multiples to derive hierarchical feature maps, facilitating detection and segmentation tasks. This approach yields three distinct types of feature maps, enhancing performance compared to relying solely on a single type of feature map. Secondly, SW-MSA (Shifted Windows Multi-Head Self-Attention) provides the model with linear complexity, reducing the computational burden and enhancing overall efficiency. Specifically, the computational complexity of both global MSA modules and window-based MSA modules for an image composed of h × w patches is as follows:


$$\begin{array}{c} \Omega (MSA)\;=\;4h\omega C^{2}\;+\;2{\left(h\omega \right)}^{2}\;C \end{array}$$ (1)

$$\begin{array}{c} \Omega (W-MSA)\;=\;4h\omega C^{2}\;+\;2M^{2}\;h\omega C \end{array}$$ (2)

where M represents the window length, while h and w denote the height and width of the input image, respectively. When M is significantly smaller than the input size, the complexity of the Swin Transformer Ω(W − MSA) can be approximated as 4hωC2, which is linear. The Swin Transformer achieves an accuracy of 84.2% on ImageNet-1K and 86.4% on ImageNet-22K, outperforming the ViT model by 6.3% and 1.2%, respectively [13]. On the COCO test-dev dataset, Swin Transformer achieves a box AP of 58.7 and a mask AP of 51.1, surpassing the state-of-the-art (SOTA) models by 2.7 and 2.6 points, respectively [13].

The RepVGG model, derived from the VGG architecture, offers a simplified yet potent alternative comprising only 3 × 3 convolutions and ReLU activation functions. RepVGG employs structural reparameterization technology to reduce the complexity during inference. The replication of layers in RepVGG helps mitigate overfitting by averaging out noise in the input data, resulting in a more resilient model that better generalizes unseen data. Additionally, by employing multiple instances of the same layer, RepVGG is able to learn diverse representations of the input data, thereby enhancing its generalization performance. The RepVGG model runs 83% faster than ResNet-50 and 101% faster than ResNet-101, while maintaining higher accuracy [14]. Moreover, the RepVGG-B2 model achieves only 58% of the parameter count, a 10% increase in speed, and a 6.57% improvement in accuracy compared to VGG-16 [14].

### Recurrent criss-cross polarized attention

The Recurrent Criss-Cross Attention (RCCA) mechanism captures long-range contextual information both horizontally and vertically, aggregating pixel-wise contextual information in a crossover fashion to yield a global feature representation of the input data. Conversely, the polarized self-attention mechanism focuses on channel and spatial dimensions while also incorporating global contextual information across horizontal and vertical axes, along with finer-grained features. RCCA uses a more streamlined cross-attention mechanism than the Non-Local attention mechanism to model each point in spatial resolution, reducing the video memory footprint by 11% and the computational effort by 85% [15]. This operation can be expressed using the following formula:


$$\begin{array}{c} \text{A}=soft\max(({\text{Qu}}^{*}\text{Ω}u{)}^{*}H^{*}W) \end{array}$$ (3)

$$\begin{array}{c} {\mathrm{\backslash bold}H}_{\mathrm{\backslash bold}u}^{\prime }=\sum_{i\in \left|\Phi_{\mathrm{\backslash bold}u}\right|}{\mathrm{\backslash bold}A}_{i,u}\Phi_{i,u}+{\mathrm{\backslash bold}H}_{\mathrm{\backslash bold}u} \end{array}$$ (4)

In formula (3), A is an attention matrix, computed by Q, K, and V. Qu is a C’ × 1 × 1 vector Calculated by Q space. Ωu is the cross vector calculated by K space, with a size of (H + W - 1) × C’. H and W are Height and width. In formula (4), A_i,u_ is the cross feature calculated for each pixel point on the H × W plane. Φ_i,u_ is a cross feature calculated by V space. H_u_ is the original input feature matrix. H’_u_ is the final output result of the module.

### Polarized Self-attention Mechanism

The polarized self-attention mechanism (PSA) effectively maintains high internal resolution through polarized filtering, aligning the output distribution with typical fine-grained regression outcomes and extracting nuanced features [16]. This approach efficiently reduces computational overhead, mitigates issues associated with exploding video memory resulting from high dimensionality, and consistently delivers commendable performance.PSA leverages Self-Attention to determine attention weights, fully exploiting the modeling capabilities inherent in Self-Attention structures. Additionally, It undergoes dimensionality reduction, ensuring efficient long-distance modeling while maintaining computational feasibility. Experimental results show that PSA improves the standard baseline by 2% on the 2D pose estimation and semantic segmentation benchmarks, and improves the state-of-the-art by 1% [16]. This operation can be expressed using the following formula:


$$\begin{array}{c} PSA_{s}\left(\mathrm{\backslash bold}X\right)\;=\;A^{sp}\left(A^{ch}(X)\right) \end{array}$$ (5)

In formula (5), A^ch^(X) represents the channel attention result of the input feature map X. A^SP^ is a function that calculates the space attention of the input data. PSA_S_(X) represents the final output result of the PSA attention module.

## Methodology

In this section, our presentation is structured into the following segments. Firstly, we introduce a novel fusion attention-based feature extraction module. Next, we delineate the fusion methods and modular structures utilized for handling multi-scale features. Finally, we elaborate on the overall structure of the fusion model.

### Faster Recurrent Criss-Cross Polarized Attention module

In this module, we leverage the RCCPA module to capture the global characteristics of the feature plane and the PSA module to extract fine-grained characteristics, integrating them into a novel feature extraction module. This approach effectively captures global characteristics across channel, spatial, and feature planes, while preserving local characteristics of the data matrix. However, the fusion of additional features increases network load and introduces feature redundancy, necessitating further dimensionality reduction beyond the 1 × 1 convolution. Given the large footprint of the RCCPA module within the integrated attention module, this paper introduces a lightweight algorithm for the RCCPA module to reduce the overall computational burden of the integrated attention module. The fused features encapsulate a broader spectrum of feature information, compensating for any missing elements and bolstering the model’s overall robustness. We call this the focus module FRCPA.

Specifically, our lightweight RCCPA feature matrix captures remote dependencies and attention in horizontal and vertical pixels in a more sparse manner. Instead of computing dependencies for each pixel point across the entire level and vertical direction, we calculate dependencies at intervals between each pixel point. This sparse calculation method significantly reduces the computational load of the module while still maintaining its ability to integrate global characteristics. The lightweight RCCA module, called FRCA, follows these steps:

1. Duplicate the original matrix H and downsample it on the feature plane by a factor of 0.5 to obtain matrix H’.
2. Apply matrix H’ to the RCCA module to calculate the attention matrix. Then, downsample the attention matrix by a factor of 2 to obtain matrix M, which has the same size as H.
3. Add matrix M to the original matrix H to produce the output of the lightweight RCCA module.

By employing sampling and upsampling, we obtain a sparser attention matrix, effectively reducing the computation and size of the model. We further compress the FRCA module and the PSA module to create the final FRCPA module. Utilizing a 1x1 convolution, we compress the dimensionality of the fused feature matrix, reducing feature redundancy and computational load without compromising performance. Therefore, the output of the FRCPA module can be expressed as a formula:


$$\begin{array}{c} FRCPA(X)={Conv}_{1*1}(PSAs(X),FRCA(X)) \end{array}$$ (6)

**Fig. 1:**
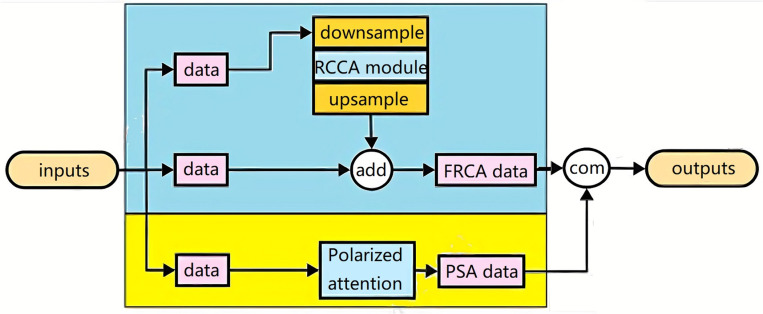
The structure of the FRCPA module.

The structure of this integrated attention mechanism can be expressed as Fig 1:

### Multi-scale strip pooling fusion module

**Fig. 2:**
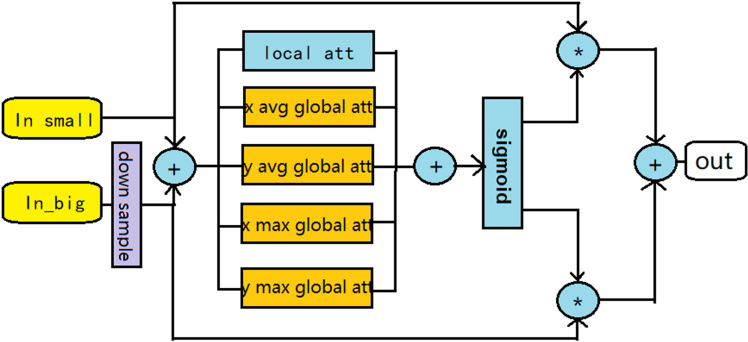
The structure of the multi-scale fusion module.

In the traditional AFF feature fusion model [17], global average pooling is used to compute global feature data, but this approach has been somewhat coarse. Strip pooling, on the other hand, captures long-range dependencies more efficiently by employing long and narrow pooling kernels (1 × N or N × 1), as opposed to the conventional N × N pooling kernels [18]. We implement a multi-branched strip pooling structure to compute global feature data, which yields more diverse global features than relying solely on average pooling. The multi-branched, multi-axis orthogonal pooling allows for a comprehensive and efficient extraction of global features from the matrix. Additionally, we incorporate downsampling to fuse features from various layers, maximizing the utility of each layer’s features, thereby accelerating the model’s convergence and mitigating gradient vanishing. The structure is illustrated in Fig 2.

### Swim-Rep fusion network

We employ a structure that serially stacks Rep blocks and Swim-Trans blocks to form the fusion network. Previous studies have demonstrated that the Swim-Transformer and RepVGG networks achieve superior classification performance. The Swim-Transformer-base model comprises four layers, containing (2, 2, 18, 2) Swim-Trans blocks, respectively, while the RepVGG_B0 model also consists of four layers with (4, 6, 16, 1) Rep blocks. Swim-Trans blocks are adept at capturing global features, whereas Rep blocks excel at extracting local features. By combining these two types of blocks, we can effectively obtain both global and local features. Specifically, we first modified parts of the Rep and Swim blocks’ structures to accommodate serial, parallel, and overlapping connections. We then adjusted the depth of each layer within the four-layer structure. In the Swim-Transformer-base and RepVGG_B0 models, most blocks are stacked in the third layer, while the other layers have relatively shallow structures, leading to inefficient feature extraction and resource allocation. To address this, we redistributed the blocks more evenly across all layers, ensuring that the model fully utilizes features from each layer, potentially enhancing overall performance. Furthermore, we added a multi-scale fusion module and an attention module between each layer to filter and enhance the features, improving the model’s convergence. The final fused features retain more information and capture superior multi-scale characteristics, ensuring that features from each scale layer are fully utilized. This results in a fusion matrix that encompasses a broader and more diverse feature set.

**Fig. 3:**
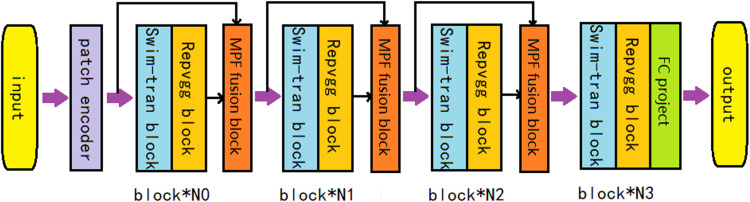
The structure of multi-scale feature fusion block.

Additionally, by integrating the FRCPA (Faster Recurrent Criss Cross Polarized Attention) module into the original data matrix, we enhance global attention across multi-dimensional characteristics, improving the capture of fine-grained features. The lightweight attention module not only boosts computational efficiency but also enhances the model’s expressive capacity. With a unified attention mechanism, our model gains a more comprehensive understanding of the input data from multiple perspectives. By integrating multi-scale features from both the Transformer and VGG models, we combine information across various levels of abstraction, enriching the overall dataset representation. Multi-scale feature fusion facilitates the extraction of features at different spatial resolutions, enabling neural networks to capture intricate details and global context simultaneously. In complex datasets, such as images, information manifests across multiple scales, ranging from low-level textures to high-level semantics. Combining features extracted at different scales promotes information flow across diverse levels of abstraction, allowing the network to capitalize on both fine-grained details and holistic context. The overall network structure is depicted in Fig 3.

## Experiments

We used two datasets in our experiments. The first is the MIT-BIH ECG classification dataset, available at https://physionet.org/content/mitdb/1.0.0/. The MIT-BIH Arrhythmia Database comprises 48 half-hour segments of dual-channel ambulatory electrocardiogram (ECG) recordings from 47 subjects. The recordings were digitized at 360 samples per second per channel with 11-bit resolution within a 10 mV range. Each record was independently annotated by two or more cardiologists. The second dataset is the RSSCN7 remote sensing map classification dataset, available at Baidu AI Studio. The RSSCN7 Dataset contains 2,800 remote sensing images across 7 typical scene categories: grasslands, forests, farmlands, parking lots, residential areas, industrial areas, and rivers and lakes. Each category includes 400 images sampled at 4 different scales. The pixel size of each image is 400 × 400, with samples taken across different seasons and weather conditions.

### MIT-BIH dataset experiment

We utilized the wfdb library to read ECG data from the MLII leads in the raw database and extracted the labeled data from the accompanying ATR files, storing it in matrix format. Identified the R-wave peak points in the heart rate annotation data. Extracted data segments consisting of 0.5 seconds before and 0.4 seconds after each peak location. Applied wavelet transform to the ECG signal to eliminate baseline drift caused by respiratory-induced electrode movement. Smoothed the data using filters to remove 60Hz power frequency interference and other artifacts. We obtained ECG signals for five types of arrhythmias: “F,” “N,” “Q,” “SVEB,” and “VEB.” The entire processed dataset was divided into two parts, with 80% allocated to the training set and 20% to the test set. The model was trained using an input size of 224 × 224, with a learning rate set to 0.0001. The cross-entropy loss function was used as the objective function for the model. To evaluate the model’s comprehensive performance, we recorded its accuracy, precision, recall, and F1 score.

### MIT-BIH semi-supervised experiment

**Fig. 4:**
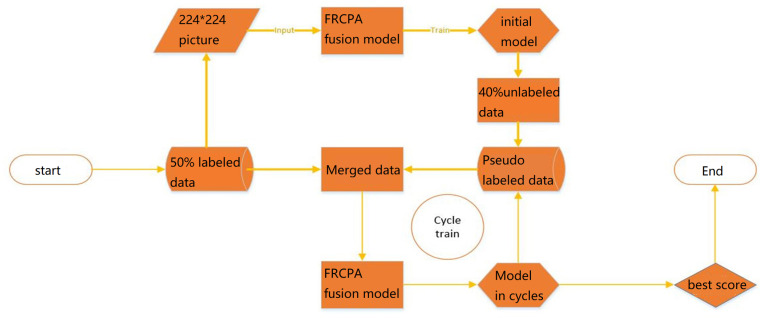
The process of training a semi-supervised model based on pseudo labels.

Repartition of the dataset based on the fully supervised experiments to create a 50% labeled training set, a 40% unlabeled training set, and a 10% validation set. Conduct semi-supervised training using a pseudo-labeled training method. The training process of a semi-supervised deep learning model using pseudo labels can be illustrated in Fig 4.

The procedure for semi-supervised training is as follows

Step 1: Train an initialized model M using 50% of the labeled dataset.

Step 2: Use model M to predict 40% of the unlabeled training set. Set the filtering confidence threshold to 0.95. Place the data with prediction accuracy greater than this confidence in the fakepic folder, and the data with lower confidence in the unfakepic folder.

Step 3: Use the pseudo-labeled data with higher prediction confidence from the fakepic folder, along with the 50% of the labeled data from the datapic folder, as the training data for the next round. Continue training model M.

Step 4: Use the model trained in the previous step to predict another 40% of the unlabeled data and repeat Steps 2 and 3.

Step 5: Repeat the process until the model’s accuracy no longer improves.

### RSSCN7 dataset experiment

We organized the downloaded dataset into appropriate folders based on categories, resized the images to 224 × 224, and normalized them. The dataset was then randomly divided into training and test sets in an 8:2 ratio. The learning rate was set to 0.0001 and adjusted according to the step sizes of 80, 160, and 240, with a scaling factor of 0.1.

To further validate the performance of different models and assess the contribution of each module, we conducted comparison experiments using six groups of models: the Swim-Transformer-base model, RepVGG-B0 model, Swim-Rep fusion model, Swim-Rep-AFF model, Swim-Rep-AFF-FRCPA model, and the Swim-Rep-MPF-FRCPA model. The last model represents the final model proposed in our paper.

## Results and discussion

**Fig. 5:**
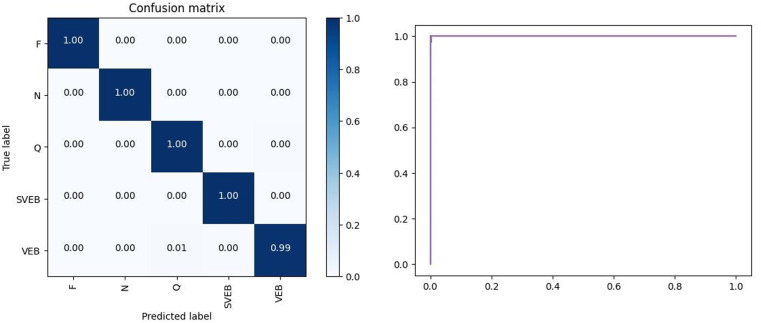
The confusion matrix and roc curves for the experiment.

Our best model, trained on the MIT-BIH dataset, achieved a top-1 accuracy of 99.82% after 20 epochs using the fully-supervised fusion model, surpassing the ViT model by 0.12%. Moreover, the fusion model demonstrated an impressive F1-score of 99.53% in SVEB classification tasks, significantly outperforming the ViT model’s F1-score of 60.2%. We have plotted the confusion matrix and ROC curves for this model at its optimum, as shown in Fig 5. These plots illustrate the model’s ability to efficiently classify various ECG data. The ROC curves indicate that the model achieves optimal classification performance, with an AUC value approaching 1.0. These results clearly highlight the superior effectiveness of the fusion model in arrhythmia classification tasks.

We conducted a comparison of the performance of our fusion model with previously studied models, assessing accuracy and algorithms across the same training set. The results are summarized in Table 1, demonstrating that our model surpasses all previous models and algorithms in terms of accuracy.

**Table 1:** Algorithms and accuracy of various previous models.

Author	DL algorithm	Accuracy
Rahhal et al. [19]	Stacked denoising auto-encoders with sparsity constraint	99.7%
Luo et al. [20]	Stacked denoising auto-encoders	97.5%
Yang et al. [21]	Stacked sparse auto-encoders	99.5%
Oh et al. [22]	Modified U-net	97.2%
Hou et al. [23]	LSTM-based auto-encoder	99.74%
Neha Shukla et al. [24]	ECG-ViT	99.7%
Ours *	Swim-Rep Fusion Net	99.82%

In the VE and SVE classification task, we conducted a comparison between the high-performing ViT model and our model in terms of F1-score and positive predicted value. The results are presented in Table 2, clearly indicating a significant performance improvement of our model over the ViT model.

**Table 2:** Our model outperforms other classifier for two classification tasks.

author	VE Positive predicted value	F1-score	SVE Positive predicted value	F1-score
Wiens and Guttag [25]	87.4	99.1	53.7	58.2
ECG-ViT [24]	89.6	99.4	57.6	60.2
Ours *	90.7	99.5	99.5	99.5

In the semi-supervised experiment, we utilized 50% of the labeled data for training. Initially, our model achieved an accuracy of 98.36% after 68 epochs, with a training loss of less than 0.001. This model was then utilized to generate pseudo-labeled data from 40% of the unlabeled data, which was combined with the labeled data for further model training. After cyclic training, we obtained a model with the highest accuracy of 98.4%. Although this accuracy is 1.46% lower than that of the fully-supervised model, it still demonstrates relatively high classification accuracy. Moreover, the model achieved a precision of 94.81% and an F1 score of 96.8% in the SVEB task, with a training loss of less than 0.03. Overall, the semi-supervised models exhibit excellent classification performance.

In the experiments on the RSSCN7 dataset, the RepVGG-B0 model, Swim-Transformer-base model, Swim-Rep fusion model, Swim-Rep-AFF model, Swim-Rep-AFF-FRCPA model, and Swim-Rep-MPF-FRCPA model achieved classification accuracies of 79.64%, 83.9%, 87.68%, 90.05%, 92.3%, and 92.5%, respectively. Their recall, precision, and F1-score in the 7-category classification task improved by 2% to 7% in the same order, as shown in Table 3.

**Table 3:** Six different models’ performances in the RSSCN7 dataset.

model	Accuracy	recall	precision	F1	Params	FLOPs
RepVGG_B0	79.64%	0.7940	0.7896	0.7915	13.1M	3.325G
Swim-transfomer-base	83.9%	0.8379	0.8383	0.8379	86.78M	15.437G
Swim-Rep fusion model	87.68%	0.8744	0.8794	0.8749	185.4M	23.78G
Swim-Rep-AFF	90.05%	0.9092	0.912	0.9099	185.9M	23.9G
Swim-Rep-AFF-FRCPA	92.3%	0.9228	0.9230	0.9226	187M	24.5G
Swim-Rep-MPF-FRCPA	92.5%	0.9251	0.9248	0.9248	189.68M	24.59G

As seen from the table above, the performance of the Swim-Rep fusion model is significantly better than that of the individual standalone models. The classification accuracy of the Swim-Rep-AFF model is 2.4% higher than that of the Swim-Rep-AFF-FRCPA model, demonstrating that the FRCPA attention mechanism significantly enhances model performance by effectively improving channel, spatial, and fine-grained feature extraction. Additionally, incorporating the FRCPA attention mechanism increases the model’s overall FLOPs by only 0.6G, resulting in a 2.3% accuracy improvement with minimal computational overhead, which is highly valuable. The Swim-Rep-MPF-FRCPA model’s classification accuracy is slightly higher than that of the Swim-Rep-AFF-FRCPA model by 0.2%, indicating that the MPF module performs comparably to the AFF module and effectively fuses multi-scale features to enhance model performance. Moreover, the MPF module adds only 0.09G more FLOPs than the AFF module, yielding a modest 0.2% accuracy improvement. However, the MPF module contains five parallel feature acquisition sub-networks compared to the AFF module’s two, allowing MPF to obtain more diverse feature maps and richer feature information. This capability becomes increasingly valuable as dataset complexity rises, enabling MPF to extract superior features and demonstrating its stronger feature extraction capability. Overall, the MPF module maintains strong application value.

## Conclusion

In this paper, we introduce a new multi-scale fusion module called MPF, which reduces feature loss and enhances model representation compared to previous multi-scale fusion modules. Additionally, we incorporate the FRCPA attention module, which effectively captures multi-dimensional cross-attention and fine-grained features. Our model achieved a remarkable accuracy rate of 99.82% on the MIT-BIH arrhythmia dataset, and achieved an accuracy rate of 92.5% on the RSSCN7 dataset. Additionally, through a semi-supervised training approach based on pseudo labels, the classification accuracy reached 98.4%. These results highlight the efficacy of our model for classification tasks and its potential application in analyzing large volumes of unlabeled medical data. In our upcoming research, we plan to explore weakly supervised models to address the challenges posed by the growing volume of medical data.
